# The Four Years’ Course from a Medical Student’s Standpoint

**Published:** 1898-04

**Authors:** J. B. Woods


### THE FOUR YEARS' COURSE FROM A MEDICAL STUDENT'S STANDPOINT.

Many of the leading medical schools now require a four-years' course of study before the student shall be graduated and receive his diploma, and many more schools, urged on by the professors, the students and the medical fraternity, are making constant efforts to "raise the standard of medical education." All this in itself is praiseworthy, and meets the hearty approval of all concerned, but there may be an honest difference of opinion as to the best method to be adopted in order to bring about this very desirable result. The reason for the necessity of this additional year's work on the part of students, with its added expense and labor, is said to be that the standard of medical attainments required has advanced so rapidly that it is impossible to cover the present field in a three-years' course, and that therefore, a four-years' course of study has become necessary.

Now the present is especially a utilitarian age, and money and labor-saving devices and methods are largely in evidence; consequently, it becomes an open question whether students should be required to lengthen their course of study when the same and even better results could be accomplished by the employment of better and more scientific methods of teaching. In mere justice to the student, should not the resources of the best educational methods be exhausted before another year is added to the curriculum, and a four-years' course of study made obligatory? It is greatly to be questioned whether the student will gain enough by this added year of labor, so long as the present system of medical instruction prevails, to at all compensate for the time and money expended; and it is certain that if the methods in vogue in other departments of educational work were given a trial in our medical schools, that the graduates after three years' study, or perhaps, even after a two-years' course, would be better prepared and equipped for their life-work.

It is matter for grave doubt whether the evils which afflict our present plan of medical education are so much due to lack of time as to faulty and inadequate methods and to the poor quality of teaching. Before the faculties of our medical schools decide that a student must spend a fourth year in training for the medical profession, should not something be done to bring medical instruction under the same wise plan which now prevails in every other department of educational work, and should not some effort be made to increase the quality as well as the quantity of the instruction? Is it not about time that something be required of the professors in our medical schools besides the fact that they are successful physicians, accomplished talkers, members of certain cliques, or animated by a due respect for the acquirement of the loaves and fishes?

All these, no doubt, are very necessary accomplishments, but should not the teacher at all worthy of the name be actuated and inspired by something different and better than all this?

According to the best exponents of that newer system of teaching of which Pestalozzi was the forerunner and founder, and which culminated in the theories of Hegel and the methods of Froebel, the basic principles upon which a successful plan of education rests, are first, interest; second, apperception; and in order that the student may get the full benefits of his labors, it is absolutely essential that these two principles should be given due consideration both in arranging the course of studies and in the methods used in conveying instruction. When these two basic principles—which have come to be considered the moving power in the modern educational system—are combined in one plan, then much has been done toward assuring the great object of education—the unfolding, the growth, the development of the human mind.

The so-called theory of apperception has worked out and is able to state the laws which govern the processes of mental assimilation: "We build the new upon the old; our grasp of a new idea is controlled by the character of the older group of ideas already existent in the mind, and all teaching must aim at preserving a certain relation between the new material offered and the present content of the mind. A study of the processes of apperception gives the death-blow to the theory that the mind can be filled with and retain isolated facts. The new idea must find itself at home, it must be at once welded to the old or it will rapidly slip away. To apperceive, however, requires mental activity, and this can be assured only when the mind occupies that attitude toward a subject which is denoted by the term 'interested.'"

Now the curriculum of our medical schools, no matter what may be urged in favor of its logical sequences, violates the law of interest at the start, for the thing which gives anatomy, pathology and therapeutics their interest in their application to medical and surgical diseases, yet the student is compelled to wrestle for two years with the hard and dry-as-dust facts of these very necessary studies unaided by that impetus which the presenting of the more interesting parts of the course would give him and uncheered to that greater assimilative power of mind which only interest in a study can produce. Not alone is the present arrangement of the course of medical studies fatal to interest, but the laws of apperception are rendered of no effect, for the benefits arising from a stimulation of the apperceptive faculties depend to a large extent upon proper correlation of facts; that is, the value of new facts depends upon perceiving their relation to other older facts. The facts of anatomy and of therapeutics become of value when closely affiliated with the treatment of medical and surgical disease, but when a delay of months or even of years intervenes between the presentation of these related subjects, a loss of the old ideas necessary for apperception results, and the student has an imperfect grasp of the new ideas. As a certain distinguished educator in treating the subject of medical instruction, has said: "Medicine is a difficult science to teach at the best, yet our medical faculties arrange a curriculum which both displays their pedagogical ignorance and adds to the labors of the student."

Another fault of the system now in use is that there is no consultation and no consequent concerted action among the professors as to how and what they shall teach. How much time in most medical schools do the different professors spend in adjusting their individual courses so that there shall be harmony in the instruction given by the various chairs. Does Professor A. ever know exactly what Professor B. is doing and saying, so that where their fields approach each other there isn't a blind path in which the poor bewildered student, now traveling awhile in this direction and now awhile in that, doesn't finally have to give up in despair, seeing no guide-board to direct his footsteps right?

The anatomy taught by the holder of that chair should be identical with the anatomy of the chair of physiology, for each of these professors must necessarily trespass somewhat upon the other's domain. The chemistry of the physiology teacher should be the chemistry of the chair of chemistry, and the same holds true of every branch taught in a medical school. Let there be no conflicting teaching among the different chairs. There is no such thing possible as slicing up the science of medicine into its component branches and have each branch stand out an isolated science in itself; but the territory of one teacher must necessarily encroach upon that of the others, more or less, and where this common ground occurs there should be no confliction. What can be more discouraging to a student than to hear from one professor that a certain thing is so and so and then hear another professor give it in a different way. Professor A., for instance, says that urea is the ultimate product of nitrogenous oxidation, while Professor B. declares that uric acid, and not urea, occupies that position in the metamorphic scale; or, for another example, the anatomy professor teaches him that the pigment in the eye belongs to the choroid, and forthwith armed with this knowledge he goes before the opthalmology professor, who "hauls him over the coals" for not knowing that the pigment belongs to the retina and not to the choroid.

The chair of surgery teaches that fixation and rest is the proper treatment for a luxated joint, but the orthopedic professor insists that fixation and rest are dangerous and that passive motion is the only safe method. So it is all the way along, one professor making one statement and another contradicting it because time has not been spent in looking over one another's work with a view to eliminating monotonous repetitions and conflicting statements, and thus lightening the burden of the overworked student.

Even in anatomy, where one would think there is little ground for dissension, different professors of the subject have different ideas, and we find discord and hobbyism. Some teachers endeavor to impress their listeners that the whole science revolves around some insignificant point to which they happen to be first to call attention, wasting hours of valuable time over a matter that ought to have been disposed of in a word, and neglecting more weighty subjects which, once passed, the student has no time to recall. Woe be unto the medical student who is forced by circumstances to complete his studies in another school. While we can understand how teachers in different colleges may differ in their theories, to differ in facts is inexcusable, for a teacher should confine himself to facts, and facts should not differ the world over. A pupil in the common schools doesn't need to learn a new kind of arithmetic every time he changes teachers, neither should a medical student be forced to learn Professor B 's therapeutics if Professor A. has already taught him all the essentials. All the antiquated notions that the effervescent science of medicine is prone to are retained by some teachers and injected into their courses and the student is expected to loyally idolize these valueless and obsolete methods or remedies as the teachers themselves do.

An old philosopher, being asked the hardest lesson he ever had to learn, replied that it was to forget those things which he had learned wrong. So the poor medical student, bemuddled with two sets of teachings on a subject sometimes gets them twisted, and instead of answering Professor A. according to Professor A.'s idea, offers him Professor B.'s teaching and gets —plucked.

But the worst feature of all is the note-taking, a system by which the brain is compelled to descend from its high throne of absorbing and assimilating the thoughts in what is being said, and becomes a mere tool to transfer the thoughts into words that it may be referred to later, in which process a goodly portion is lost, and the result is often a meaningless jumble, for only a very few indeed are capable of taking readable notes. While the professors one and all condemn the habit they *compel* students to practice it. And why? Because the so-called didactic lecture is the skimmings of the various textbooks, is often a Babel of authors intended for a pie, but quite as often it proves only a hasty pudding that gives the student a wind colic. Now let us suppose that a student endeavors to follow the advice of his teacher and takes no notes, but gives his whole attention to the lecture and goes home. He revolves the subject in his mind, and as he does so he perceives portions, here and there, whose details he can not fully remember; then he consults his text-book (the one recommended by his teacher), and to his disappointment and disgust he can not find in it what he is searching for, or if he be fortunate to find it as likely as not there is a disagreement between the teacher and the text-book. He knows that the professor will insist on questions being answered only according to his own individual teaching, hence what is there left for the poor student but to get a note-book and become the disciple of the note-book fiend. All this could be remedied by allowing the didactic lecture to die the death it is destined, substituting for such a worn-out apology for imparting knowledge, the modern methods employed in other branches. But the didactic lecture in spite of all its infirmities will die a hard death, for the modern spirit of commercialism, which bolsters up its tottering form, is a factor too powerful to be downed by simple condemnation. The opportunity for self-aggrandizement and pecuniary gain, consequent upon the occupancy of a medical chair, are too tempting for the ordinary man and he never wastes any time or experiences any remorse of conscience in accepting a position for which he is in no way adapted. Let but the medical chair be stripped of its pecuniary emoluments and the holder thereof be actuated solely by a desire to impart knowledge, then medical schools would die off like house-flies with the approach of autumn.

Following the formula laid down by one of the best of our Maine educators in speaking on a kindred subject, it might be stated that the medical school exists solely for the good of the students, and that outside of this function there is no good reason for its existence; and yet, when we come to analyze the methods of instruction in vogue, we are almost forced to conclude that the present system was inaugurated and has been kept up because it was solely for the interest of the professors. Little can be aid in favor of the didactic lecture system except that it is a lazy man's method and a time and labor-saving device for the teacher. By this plan the professor is able to cover a course in hours, which, if properly presented by clinical instruction, would require weeks. It is a good plan for that sort of teacher who is so indifferent to his high calling that all he thinks of is getting his work done in the shortest and easiest way, but why should the student be expected to give time and money for the mere hearing of facts which he can glean for himself from any well-written textbook? Telling facts can hardly be dignified by the title of teaching, and such hurling of repeated volleys of facts at the students' heads, in hit-or-miss fashion, is hardly in accordance with modern ideas of instruction.

The didactic lecture is in no way conducive to the development of thoughtfulness and reflection, and it fails signally to arouse that mental activity and interest upon which a teacher's real success always depends. It is certain that if the faculties of our schools have nothing to offer the student in compensation for a fourth year of study except another year's time spent in listening to didactic lectures that the best interest of the students will be subserved by reforming the methods of imparting instruction rather than by prolonging the course.

In the recitation system the student commits to memory a certain number of pages of a text-book and the recitation which follows is, in the hands of most teachers, little more than a test of how many of the facts the student has retained. This plan is a great improvement on the didactic lecture system in that direct cramming is better than that which comes indirectly through the note-book, but nevertheless most recitations as now conducted are merely wretched substitutes for proper educational exercises. Medical examinations, the crowning feature of the whole system, are simply memory tests, and "plugging" at quiz-compends arms the student with a large number of facts which he is prepared to shoot at his inquisitors.

The whole system of medical instruction as now employed is a mere filling-up process, wherein the brain is looked upon as a mere passive, receptive organ yearning to be filled, and the much more important fact that education should serve to unfold and develop the mind is lost sight of. Cramming a mind with ill-digested and soon-forgotten facts may be a masterly feat in mental drudgery, but such mere "grinding" can hardly be called an educational process. The recitation in the hands of a trained and well-equipped teacher can be made a source of great profit to the students, but not so long as it serves merely as means to extract a lot of memorized facts. A receptive mind merely stuffed with facts by no stretch of imagination can be called an educated mind.

The two most important functions which the physician is called upon to perform are making a diagnosis and the application of therapeutic measures to the cure of disease. The methods at present employed in the medical schools give the students little insight into the applied science of either of these branches of medicine. Many students are graduated every year who have never seen, all through their college course, a single case of any one of the acute diseases, and their knowledge of applied therapeutics is limited to hearing the treatment which some professor gives to such chronic cases as attend the so-called clinics. The very things which the physician most needs after he gets into practice are grossly neglected in the present system of medical instruction. Medicine as it ought to be taught is seen only in the special courses furnished by our post-graduate schools, and it is needless to say that if medicine were taught in anything like an adequate manner in our medical schools there would be little need for so many post-graduate schools with their hosts of teachers and legions of students hungering and thirsting for true medical knowledge. Whatever may be said as to the value of the work which the post-graduate schools are doing, the fact can hardly be gainsaid that if our medical schools did their work as it can and ought to be done the demand for post-graduate instruction would not be so urgent. The mere fact of the increasing number of these schools is in itself a striking commentary on the whole system of medical education.

When methods calculated to arouse and hold the interest and to kindle and stimulate the apperceptive faculties have taken the place of the curriculum as at present arranged, when by reason of natural abilities and proper training, professors in medical schools havecome into some fitness for their work as instructors, when the clinical lecture shall have superseded the obsolete didactic plan, when students shall be given some opportunity to diagnose and study diseases as seen in patients rather than as presented in text-books, when recitations, instead of being parrot-like performances, shall be so conducted as to awaken and stimulate thoughtfulness, perception and reasoning power, and when all the methods of medical teaching when conbined shall result in "producing clearness, quickness and soundness of observation, a mind capable of retaining and readily recalling what it has learned, with power to combine and use what it knows, able to grasp the general principles which underlie particular cases, rapid and sagacious in drawing inferences, sound and wise in reasoning, full of interest and pride in its work, moved by a sense of what is good and true, energetic, honest and strong in purpose," when all these desirable and not impossible results have been achieved, then, indeed, will the professors have done their full share towards lightening the burdens of the students, and then, indeed, will the quality of medical instruction have become so improved that there will be no need, at present at least, for increasing the quantity of medical teaching, and a three-years' course will be found ample to fit the student for his work and will result in making him a credit to his college, his instructors and his profession. In short, when the professors do something like the duty, which, in the light of modern educational methods, they owe to themselves, to their calling, to the profession and to the students, then, indeed, would it seem to be time enough to inquire whether the quantity as well as the quality of medical instruction needs to be increased.

If it indeed be true that the requirements of a modern medical education have become so vast that the faculties of our schools feel warranted in demanding of students another year of time, study and tuition, then surely the students, in their hard struggle to reach such a high goal, have a right to demand from their instructors the very best system of instruction which modern educational science has been able to devise and to expect that each individual professor shall not alone be a professor, but also a teacher, armed with all the natural ability, training and zeal which that term implies and which the vocation now demands, so that he shall be so devoted to his duties as instructor that nothing in the way of other engagements, lack of time, commercialism, self-aggrandizement, indifference or indolence shall stand in the way of his giving to the students the best medical education possible, and if the professor falls short in any of these very necessary qualifications and attempts to excuse his shortcomings by relating legends as to how busy he is, and that, consequently, he can not, for the remuneration offered, take sufficient time to fit himself as a modern teacher or to impart instruction in a proper way; then, indeed, how can such a poorly-endowed professor have the assurance to ask students to devote more time to their studies, when he himself so signally fails in the duties which he owes to himself, to his vocation and to his school.

The writer wishes to acknowledge his indebtedness in preparing this paper to Professor Richard G. Boone, of the Michigan State Normal School, to Professor Montgomery A. Crockett, of the University of Buffalo, N. Y., and to the *Educational Review*.—J. B. WOODS, M. D.—*Jour. of Med and Science.*

